# Analysis of Chromatin Accessibility Changes Induced by BMMC Recognition of Foot-and-Mouth Disease Virus-like Particles through ATAC-seq

**DOI:** 10.3390/ijms242317044

**Published:** 2023-12-01

**Authors:** Weijian Han, Junjuan Zhang, Mingzhu Li, Manxin An, Limin Li

**Affiliations:** College of Veterinary Medicine, Hebei Agricultural University, Baoding 071000, China; hwj3503@163.com (W.H.); zhangjunjuan04@163.com (J.Z.); 20202200518@pgs.hebau.edu.cn (M.L.); 20217201461@pgs.hebau.edu.cn (M.A.)

**Keywords:** bone marrow-derived mast cells, ATAC-seq, mannose receptors, virus-like particles, cytokines expression, foot-and-mouth disease

## Abstract

Mast cells can recognize foot-and-mouth disease virus-like particles (FMDV-VLPs) via mannose receptors (MRs) to produce differentially expressed cytokines. The regulatory role of chromatin accessibility in this process is unclear. Bone marrow-derived mast cells (BMMCs) were cultured, and an assay of transposase-accessible chromatin sequencing (ATAC-seq) was applied to demonstrate the regulation of chromatin accessibility in response to the BMMCs’ recognition of FMDV-VLPs. A pathway enrichment analysis showed that peaks associated with the nuclear factor-κB (NF-κB), mitogen-activated protein kinase (MAPK), phosphatidylinositol 3 kinase-protein kinase B (PI3K-Akt), and other signaling pathways, especially the NF-κB pathway, were involved in the BMMCs’ recognition of VLPs. Moreover, transcription factors including SP1, NRF1, AP1, GATA3, microphthalmia-associated transcription factor (MITF), and NF-κB-p65 may bind to the motifs with altered chromatin accessibility to regulate gene transcription. Furthermore, the expression of NF-κB, interleukin (IL)-9, tumor necrosis factor (TNF)-α, and interferon (IFN)-γ in the BMMCs of the VLP group increased compared with that of the BMMCs in the control group, whereas the expression of IL-10 did not differ significantly between groups. After inhibiting the MRs, the expression of NF-κB, IL-9, TNF-α, and IFN-γ decreased significantly, whereas the expression of IL-10 increased. The expression of MAPK and IL-6 showed no significant change after MR inhibition. This study demonstrated that MRs expressed on BMMCs can affect the NF-κB pathway by changing chromatin accessibility to regulate the transcription of specific cytokines, ultimately leading to the differential expression of cytokines. These data provide a theoretical basis and new ideas for the development of a novel vaccine for FMD.

## 1. Introduction

Foot-and-mouth disease (FMD), caused by foot-and-mouth disease virus (FMDV), is an acute and highly contagious disease that can result in substantial economic losses for the livestock industry worldwide and seriously affect the development of international trade [1,2,3]. Virus-like particles (VLPs) present a promising vaccine candidate and have attracted increasing attention from many researchers who expect that they will play a vital role in global FMD control [4,5,6,7,8]. The pharyngeal region, which is the primary site of FMDV replication during acute infection, can excrete low levels of the virus asymptomatically during persistent infection [9,10]. Strategies to eliminate the virus more efficiently at the primary site of infection are crucial for the development of novel FMD vaccines.

Mast cells (MCs), a type of sentinel cells, are strategically located in tissues near the external environment. MCs, macrophages, and dendritic cells comprise the first line of defense against invading pathogens [11]. MCs are best known as key effector cells of IgE-mediated allergic responses. However, MCs can be activated when invading pathogens can be recognized by a wide array of pattern-recognition receptors (PRRs). Activated MCs can immediately release a unique panel of biologically active mediators, regulating innate immunity and adaptive immunity against bacterial and viral infection [12,13,14]. Our previous study demonstrated that MCs can recognize FMDV-VLPs or the recombinant FMDV VP1-VP4 protein to secrete various differentially expressed cytokines via mannose receptors [15,16]. Compared with the control group, the levels of IL-1α, IL-2, IL-4, IL-15, IL-17A, IL-21, TNF-α, IFN-γ, CCL-17, and CCL21 of the VLP group were all significantly upregulated (*p* < 0.05) and IL-10 showed no difference, while the levels of IL-1α, IL-2, IL-4, IL-15, IL-17A, IL-21, TNF-α, IFN-γ, CCL-17, and L-selectin of the iMR-VLP group were significantly downregulated (*p* < 0.05). It is noteworthy that the expression of IL-9, IL-10, CCL19, and CCL21 were further upregulated in the iMR-VLP group [15].

Evidence has indicated that epigenetic changes, such as alterations in chromatin structure and DNA base modifications, play a key role in gene regulation mechanisms [17,18]. During DNA replication and transcription, some regions of chromatin are opened, and transcription factors can bind to the exposed DNA sites in open regions to initiate and regulate DNA replication or transcription [19]. Understanding the epigenetic state of chromatin in a certain biological context can elucidate the molecular mechanisms underlying the observed gene expression patterns [20]. Previous studies have applied an assay of transposase-accessible chromatin sequencing (ATAC-seq), which uses Tn5 transposase to sequence open chromatin regions in the genome to examine dynamic changes of chromatin structure [21,22,23].

Therefore, this study explores the effect of chromatin accessibility on the regulation of cytokine responses related to mast cells’ recognition of FMDV-VLPs via MRs, thereby providing a theoretical basis and new ideas for the development of a novel vaccine against FMD based on MCs.

## 2. Results

### 2.1. Bone Marrow-Derived Mast Cells Preparation

**Figure 1:**
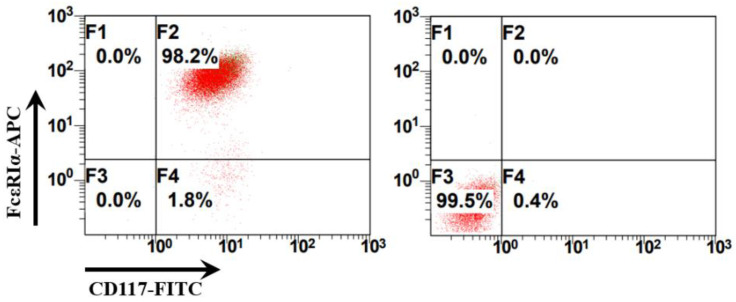
Identification of bone marrow-derived mast cells (BMMCs) using flow cytometry. The right panel presents the results of the isotype control antibodies. Allophycocyanin (APC) Armenian Hamster IgG isotype control antibodies (BioLegend, San Diego, CA, USA) and fluorescein isothiocyanate (FITC) Rat IgG2b, κ isotype control antibodies were used as isotype controls (BioLegend, San Diego, CA, USA). The left panel presents the results of the BMMCs labeled with APC Armenian Hamster anti-mouse FcεRIα antibodies (IgG) and FITC Rat anti-mouse CD117 (c-Kit) (IgG2b, κ) antibodies and identified using flow cytometry.

**Figure 2:**
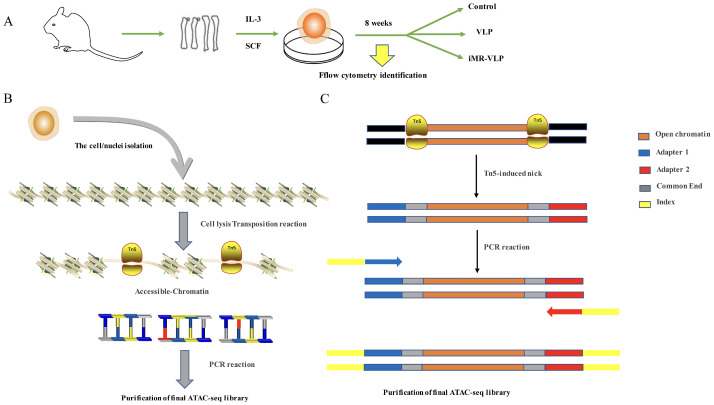
Schematic of the assay of transposase-accessible chromatin sequencing (ATAC-seq) transposition reaction and library preparation. (**A**) BMMCs collected for ATAC-seq comparison. (**B**) Overview of the steps in ATAC-seq. The nuclei are isolated from the cells, keeping the chromatin structure intact. This chromatin is then exposed to Tn5 transposase. Only fragments with adapters can be properly amplified and sequenced. (**C**) Detailed schematic of fragments generated by transposition into native chromatin. After Tn5 insertion of the polymerase chain reaction (PCR) handles, the nicks left behind by transposase are filled in during the initial 72 °C extension in the first step of the barcoding PCR. Subsequently, the fragments receive Adapter 1 at one end and Adapter 2, and they then are barcoded and prepared for sequencing.

Bone marrow cells were collected from the femurs of C57BL/6N mice and cultured in a complete RPMI 1640 medium with recombinant murine IL-3 (10 ng/mL) and SCF (20 ng/mL) to obtain bone marrow-derived mast cells (BMMCs). Mast cells are c-Kit^+^FcεRI^+^ cells that originate from mast cell progenitors that are produced in the bone marrow. BMMCs were identified using flow cytometry with allophycocyanin (APC)-labeled anti-mouse FcεRI antibodies and fluorescein isothiocyanate (FITC)-labeled anti-mouse CD117 (c-Kit) antibodies. The percentage of BMMCs (c-Kit^+^FcεRI^+^ cells) can reach 98.2% in BMMC culture (Figure 1). The identified BMMCs were treated as different groups and used for the subsequent ATAC-seq sample preparation (Figure 2).

### 2.2. ATAC-seq Data Quality Control

**Figure 3:**
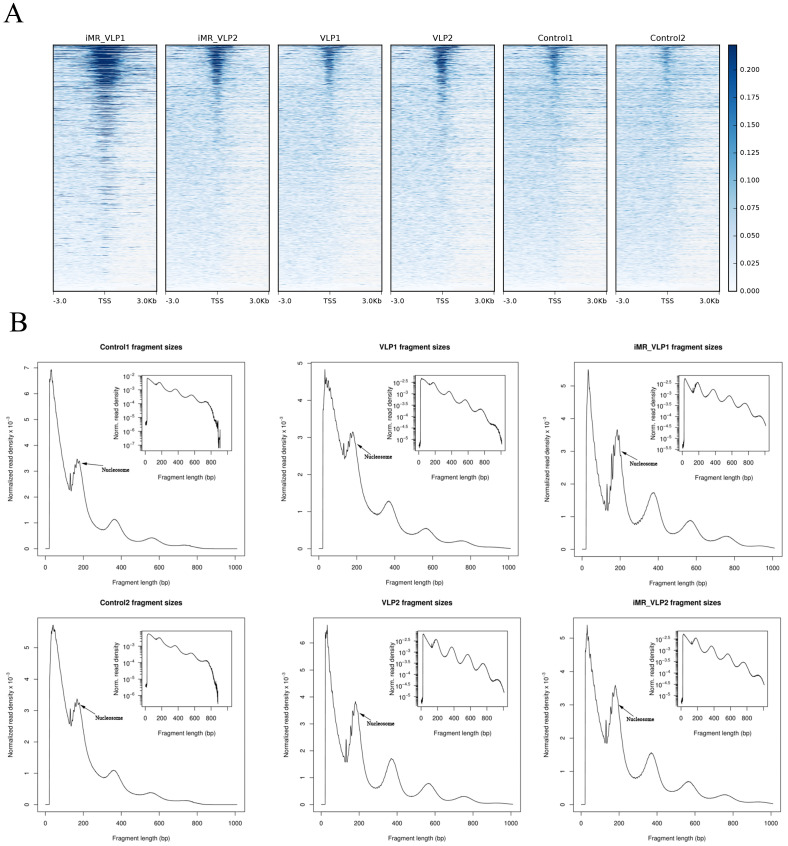
ATAC-seq library quality metrics. (**A**) ATAC-seq signal enrichment around the transcription start sites (TSSs) for all samples (with two biological replicates). (**B**) Fragment size distributions derived from the corresponding sequencing data.

We used Burrows Wheeler Aligner for a comparative analysis between the ATAC-seq dataset and mouse reference genomes. We evaluated the ATAC-seq dataset with several common statistical methods (Table 1). For each sample, an average of 50 million clean reads were obtained. The data for Unique_Mapped (the number of reads with unique mapped positions in the reference sequence) suggested that the library complexity was >65.72% in the VLP group, >71.59% in the inhibited mannose receptor (iMR-VLP) group, and >82.61% in the control group. Considering that there may be multiple reads at a specific position, the number of reads excluding multiple Unique_mapped reads (Unique_mapped_dedup) showed >50.30% in the VLP group, and >55.01% in the iMR-VLP group. The Unique_mapped_dedup reads were used for further analysis. The accessible regions were successfully detected by the observation of a strong enrichment of ATAC-seq reads around the transcription start sites (TSSs, ±3 kb) (Figure 3A). Additionally, all ATAC-seq libraries yielded the expected distribution of fragment lengths. Most fragments were small, representing internucleosome open chromatin; and only a few fragments were large, spanning the nucleosome (Figure 3B). Taken together, these results demonstrated that the ATAC-seq data were acceptable.

### 2.3. Chromatin Accessibility Profiles in BMMCs upon Their Recognition of VLPs

**Figure 4:**
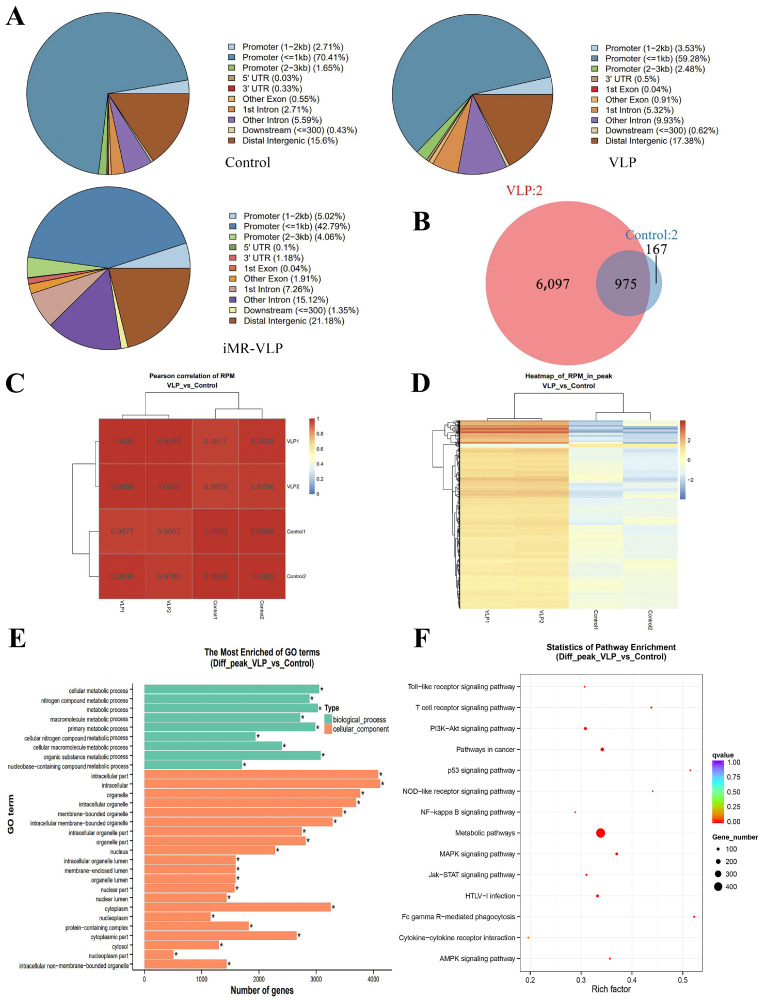
Properties of accessible chromatin regions identified in BMMCs incubated with foot-and-mouth disease virus-like particles (FMDV-VLPs). (**A**) Pie charts showing the distribution of accessible regions identified from the ATAC-seq results using ChIPseeker software (version 1.16.1) in each functional area. (**B**) Venn diagram showing an overlap of differential peak linkages identified in VLP versus control data. (**C**) Heatmap clustering of correlation coefficients across four replicates. (**D**) Hierarchical clustering heat map showing differential peaks in different samples. (**E**) The most enriched gene ontology terms for differential peaks from BMMCs in VLP vs. control samples. * *p* < 0.05. (**F**) Kyoto Encyclopedia of Genes and Genomes (KEGG) analysis of different peaks from BMMCs in VLP vs. control samples.

We assessed chromatin accessibility changes in the BMMCs incubated with FMDV-VLPs in vitro by comparing them with the control BMMCs. More than 50% peaks (≤1 kb) were located in the promoter region in both the VLP group and the control group (Figure 4A). To identify the accessible regions that were biased toward the control, VLP, and iMR-VLP groups, we first focused on a comparison between the control and VLP groups.

Approximately 6097 accessible peaks were VLP-specific, and 975 overlapped with the accessible peaks observed when the BMMCs were inoculated with VLPs (Figure 4B). A heatmap clustering analysis of the Pearson correlation coefficients from the comparison of four datasets (two biological replicates of the control group and the VLP group) revealed a strong correlation between the replicates (Figure 4C). Compared with the control group, most peaks in the VLP group were specific and upregulated (Figure 4D). A gene ontology analysis showed that the accessibility of chromatin regions containing genes related to biological processes and cell components differed greatly between the groups. Compared with the control group, approximately 16,000 related peaks were upregulated, and about 2300 related peaks were downregulated in the VLP group (Figure 4E). A pathway enrichment analysis showed that the peaks associated with the NF-κB, MAPK, PI3K-Akt, and other signaling pathways were upregulated in the VLP group, compared with the control group (Figure 4F).

**Figure 5:**
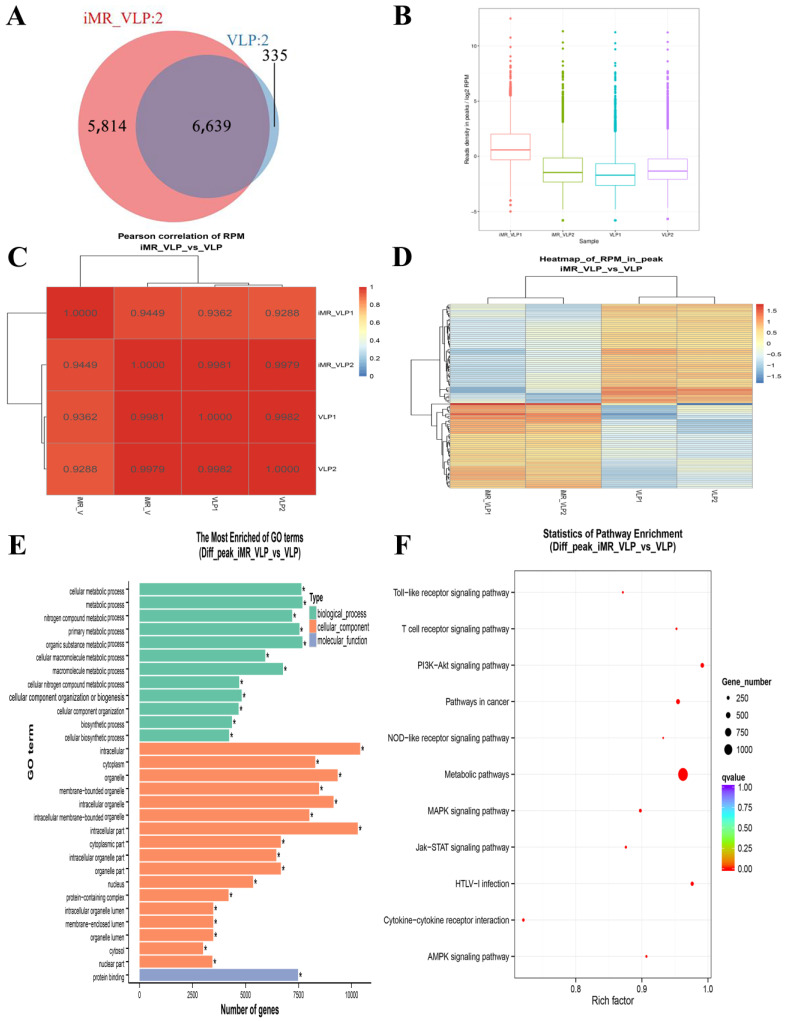
Properties of accessible chromatin regions identified in BMMCs pre-treated with inhibitors for mannose receptors (MRs). (**A**) Venn diagram showing overlap of differential peak linkages identified in inhibited mannose receptor (iMR-VLP) versus VLP data. (**B**) Reads per million (RPM) values of iMR-VLP and VLP samples. (**C**) Heatmap clustering of correlation coefficients across four replicates. (**D**) Hierarchical clustering heat map showing differential peaks in iMR-VLP and VLP samples. (**E**) The most enriched gene ontology terms for differential peaks from BMMCs in iMR-VLP vs. VLP samples. * *p* < 0.05. (**F**) KEGG analysis of different peaks from BMMCs iMR-VLP vs. VLP.

After the MRs were inhibited, the further chromatin accessibility changes were assessed. We observed 6639 overlapping peaks between the iMR-VLP and VLP groups (Figure 4B and Figure 5A). Compared with the VLP group, approximately 22,000 related peaks were upregulated, and approximately 3,400 related peaks were downregulated (Figure 5E). Many peaks were associated with metabolism. A pathway enrichment analysis indicated that the peaks associated with the NF-κB signaling pathway were downregulated, while the peaks associated with the MAPK and PI3K-Akt signaling pathways were upregulated (Figure 5F). 

### 2.4. Motif Enrichment Analysis

To obtain insight into the transcription factors governing the gene expression, we performed a motif enrichment analysis. We examined whether different peak regions were enriched for particular transcription factor binding sites in BMMCs upon their recognition of VLPs. The motif represents the sequence conservation of the peak location, which may play a role in gene expression regulation. Motifs with lengths of 8–14 bp were predicted from the differential peaks with HOMER software (version 4.9.1). The results of the HOMER de novo motif enrichment levels analysis revealed that over 23 motifs were upregulated and over 22 motifs were downregulated when the BMMCs were incubated with FMDV-VLPs (VLP group vs. control group) and when their MRs were inhibited before incubation with FMDV-VLPs (iMR-VLP group vs. VLP group), respectively. The 15 most significantly enriched motifs (according to *p*-value) are listed in Table 2 and Table 3. Compared with the control group, the enrichment levels of the motifs recognized by transcription factors, such as SP1, NRF1, AP1, GATA3, and MITF, were upregulated in the VLP group (Table 2). By contrast, the enrichment levels of the motifs recognized by transcription factors, such as RUNX1, GFI1b, NFkB-p65, and PB0126.1_Gata5_2, were downregulated in the iMR-VLP group compared with cells in the VLP group (Table 3).

### 2.5. Validation of the Expression Levels of Critical Cytokine Genes Using Quantitative Reverse Transcription Polymerase Chain Reaction Tests

**Figure 6:**
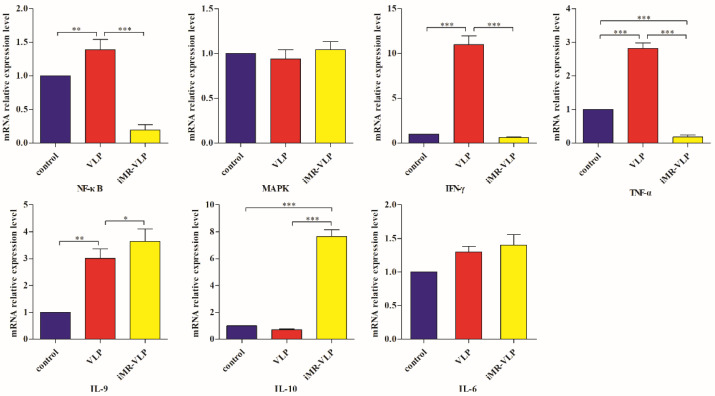
Relative mRNA levels of transcription factors and cytokines in different treatment groups. Control, BMMCs. VLP, BMMCs incubated with FMDV-VLPs. iMR-VLP, inhibited MRs then incubated with FMDV-VLPs. * *p* < 0.05, ** *p* < 0.01, *** *p* < 0.001.

We used quantitative reverse transcription polymerase chain reaction (RT-qPCR) tests to verify the expression levels of *NF-κB*, *MAPK*, *TNF-α*, *IFN-γ*, *IL-9*, and *IL-10* in the BMMCs in different groups. Compared with the control group, the expression of *NF-κB*, *IL-9*, *TNF-α*, and *IFN-γ* increased in the BMMCs of the VLP group, and the expression of *IL-10* did not differ significantly between the two groups. After inhibiting the MRs, the expression of *NF-κB*, *TNF-α*, and *IFN-γ* decreased significantly (*p* < 0.01), while the expression of *IL-10* increased. The expression of *MAPK* and *IL-6* did not differ significantly between the groups (Figure 6).

## 3. Discussion

Eukaryotic transcriptional regulation results from the interaction between many cis-regulating elements and trans-acting factors. The trans-acting elements with regulatory functions participate in eukaryotic transcriptional regulation by binding to the open chromatin region. Once a transcription factor binds to the open chromatin region, it recruits other factors to initiate gene transcription. This binding to the genome exposes the DNA, making the chromatin accessible [24]. Cytokines secreted by activated MCs play an important role in immune regulation, and cytokine expression is related to chromatin accessibility. The present study used sensitive ATAC-seq technology to characterize the changes of chromatin accessibility to reveal the gene expression regulation mechanism when BMMCs recognize FMDV-VLPs.

In combatting FMDV infection, IFN-γ is responsible for regulating the immune response against FMDV and inhibiting FMDV replication, and TNF-α can activate T cells via the Th1 pathway, promoting cellular immune responses [25,26]. The concentration of IL-10, an inhibitory cytokine, increased 3 and 4 days after FMDV infection in swine serum, suggesting that it is related to immunosuppression caused by FMDV [27]. IL-9, a cytokine mainly produced by Th9 cells, but also produced from mast cells, plays an important role in the expansion, accumulation, activation, and functions of mast cells [28,29]. Our previous study revealed that FMDV-VLPs promoted IFN-γ secretion through a mechanism mediated by MRs expressed on BMMCs and effectively inhibited IL-10 expression [15]. In the present study, the ATAC-seq analysis indicated that most genes related to the NF-κB, MAPK, and PI3K-Akt pathways were upregulated in the VLP group compared with the control group. Once the MRs were inhibited, genes associated with the NF-κB pathway were downregulated, whereas genes related to the MAPK and PI3K-Akt pathways were upregulated (Figure 5). The important transcription factor NF-κB promotes the secretion of inflammatory cytokines, such as IL-1β, IFNα, IFNβ, IFNγ, and TNFα through the activation of the inflammasome. The relative mRNA level of NF-κB in the VLP group was increased compared with the control group, and similar trends were observed for IFN-γ, TNF-α, and IL-9. These results suggest that changes in chromatin accessibility may be connected to the differential expression of the cytokines we evaluated previously. 

Transcription factors can recognize and bind to DNA regions (motifs) with sequence specificity to regulate nearby gene transcription. Generally, when chromatin is more open, transcription factors can more easily bind to DNA [30]. Therefore, analyzing the accessibility of de novo transcription factor binding motifs is essential to identify the transcription factors that regulate gene transcription when BMMCs recognize VLPs. In our study, the enriched motifs bound to several transcription factors that regulate gene expression (SP1, NRF1, AP1, GATA3, MITF, etc.) were upregulated in the VLP group compared with the control group. Specificity protein 1 (SP1), a well-known transcription factor, can activate the transcription of many cellular genes that contain putative CG-rich SP-binding sites in their promoters, such as IL-10 [31]. Transcription factor clusters consisting of NFATC2, STAT5, GATA2, AP1, and RUNX1 binding sites in the proximal IL-13 enhancer are critical in the presence of mouse MCs to antigenic stimulation [32]. Therefore, the increased expression of TNF-α, IFN-γ, and IL-9 may be related to the transcription factors listed in Table 3. However, further studies are needed to determine which transcription factors control the corresponding cytokines.

Compared with the VLP group, the binding motifs of several transcription factors (RUNX1, NF-κB, etc.) were downregulated in the iMR-VLP group. DNA-bound NF-κB can interact with many transcription factors to orchestrate the timing and amplitude of gene expression [33]. The results of the real-time PCR testing indicated that BMMCs not only affect chromatin accessibility but may also influence the NF-κB pathway via MRs to regulate the expression of downstream cytokines when the cells recognize VLPs (Figure 6). A limitation of our study is that we did not validate all the transcription factors that bind to enrichment motifs, and therefore, we could not determine more detailed mechanisms related to the BMMCs’ recognition of FMDV-VLPs.

## 4. Materials and Methods

### 4.1. Mice

Six- to eight-week-old SPF C57BL/6N male mice (certificate no.:110011210111475962) were purchased from Beijing Vital River Laboratory Animal Technology Co., Ltd. (Beijing, China). All relevant procedures involving mice followed the Laboratory Animal Guidelines of the Ethical Review of Animal Welfare in China (GB/T35892-2018) [34] and were approved by the Animal Welfare and Ethics Committee at the Laboratory Animal Centre of Hebei Agricultural University (approve code: 2019006). 

### 4.2. Cell Samples Preparation

The mice were sacrificed by cervical dislocation. The BMMCs were obtained as described previously [16,35]. The BMMCs were cultured in a complete RPMI 1640 medium (Gibco, Grand Island, NY, USA), and supplemented with 10% fetal bovine serum, 100 U/mL penicillin/streptomycin (Gibco, Grand Island, NY, USA), 10 ng/mL recombinant murine IL-3 (rIL-3, Novoprotein, Suzhou, China), and 20 ng/mL recombinant murine stem cell factor (SCF) (rSCF, Novoprotein, Suzhou, China) at 37 °C under 5% CO_2_. After 8 weeks, the BMMCs were identified using a flow cytometric analysis of the expression of c-Kit (CD117) (BioLegend, San Diego, CA, USA) and FcεRIα (BioLegend, San Diego, CA, USA). 

Approximately 5 × 10^5^ BMMCs/well were seeded into 24-well cell plates (1 × 10^6^ BMMCs/mice). The BMMCs were divided into three groups: (1) BMMCs pre-treated with MR inhibitor (mannan with a final concentration of 3 mg/mL) for 2 h before incubation with FMDV-VLPs (iMR-VLP group), (2) BMMCs incubated with FMDV-VLPs (VLP group), and (3) BMMCs only (control group). Two biological replicate experiments were performed in each group. FMDV-VLPs with a final concentration of 20 μg/mL were added to corresponding groups and the cell plates were cultured at 37 °C in a 5% CO_2_ incubator. After incubation for 24 h, the BMMCs were collected, washed, and frozen in liquid nitrogen.

### 4.3. Library Construction and Sequencing

The cell viability of the BMMCs was identified, and ATAC-seq library preparations were carried out as previously reported [21]. The detailed schematic of the cell preparation and library construction is shown in Figure 1. Briefly, the BMMCs were washed with phosphate-buffered saline (PBS) after centrifugation, and then the cells were lysed using a cold lysis buffer and centrifuged again. The nuclei pellet was resuspended in the Tn5 transposase reaction mixture and incubated for 30 min at 37 °C. Subsequently, the DNA product was purified using a Qiagen MinElute Purification Kit (Shenzhen, China). Equimolar Adapter 1 and Adapter 2 were added after the transposition, and the genes were then amplified for the full library. After the PCR amplification, the libraries were purified with the AMPure beads, and the quality of the libraries was assessed with Qubit. The clustering of the index-coded samples was performed on a cBot Cluster Generation System with a TruSeq PE Cluster Kit v3-cBot-HS (Illumina, San Diego, CA, USA) according to the manufacturer’s instructions. The library preparations were then sequenced on an Illumina Hiseq platform, and 150 bp paired-end reads were generated. The ATAC-seq analysis was carried out at Novogene (Tianjin, China) with two biological replicates per group.

### 4.4. Validation of the Expression Levels of Critical Cytokine Genes Using RT-qPCR Tests

To further verify the expression levels of critical cytokine genes, BMMCs samples were divided into the three groups as described above (the iMR-VLP group, the VLP group, and the control group). The total RNA was extracted with TransZol Up (TransGen, Beijing, China), followed by reverse transcription with the M-MLV reverse transcriptase (Promega, Beijing, China) to obtain the cDNA. Subsequently, the cDNA was utilized to amplify the critical genes with three-step RT-qPCR tests. The primers were designed according to the coding sequence (CDS) of the following genes: *NF-κB* (AY388959), *MAPK* (X58712), *TNF-α* (BC137720), *IFN-γ* (BC119063), *IL-9* (NM_008373), and *IL-10* (M37897). The qPCR reaction mixture comprised 10 μL PerfectStart Green qPCR SuperMix (TransGen, Beijing, China), 1 μL of 10 μM forward/reverse primers, 2 μL of cDNA, and a volume of ddH_2_O to obtain a final volume of 20 μL. The details of primers used in this study are listed in Table 4.

### 4.5. Statistical Analysis

The 2^−ΔΔT^ method was used to analyze the relative content of mRNA in different groups. GraphPad Prism 9.0 was used for the data analysis and a one-way analysis of variance (ANOVA) was used to compare the relative gene expression between groups. Differences with a *p*-value of *p* < 0.05 were considered statistically significant.

## 5. Conclusions

This study demonstrated that the MRs expressed on BMMCs can affect the NF-κB pathway by changing chromatin accessibility to regulate the transcription of specific cytokines, such as IL-9, TNF-α, IFN-γ, and IL-10, ultimately leading to the differential expression of cytokines. Due to the important role of these cytokines in immune regulation and antiviral infection, as well as the unique position of mast cells as sentinel cells, we can focus on the regulation of dendritic cell antigen presentation function by mast cells and the immunomodulatory effect of mast cells on macrophages in vitro or in vivo. Therefore, novel vaccines (including novel adjuvants) can target mast cells in the future. These data elucidate one of the mechanisms by which mast cells recognize foot-and-mouth disease virus-like particles to secrete cytokines differentially and provide a theoretical basis and new ideas for the development of a novel MC-based FMD vaccine.

## Figures and Tables

**Table 1:** ATAC-seq metadata and mapping statistics.

Sample	Clean_Reads	Mapped_MT	Mapped_non_MT	Unique_Mapped	Unique_Mapped_Dedup
Control1	50,330,055	742,506 (1.48%)	46,268,347 (91.93%)	41,579,440 (82.61%)	32,311,000 (64.20%)
Control2	49,614,421	514,427 (1.04%)	46,851,917 (94.43%)	42,269,831 (85.20%)	32,833,186 (66.18%)
iMR_VLP1	58,585,184	6,230,516 (10.63%)	47,602,533 (81.25%)	42,405,455 (72.38%)	31,648,544 (54.02%)
iMR_VLP2	50,138,881	2,279,086 (4.55%)	40,804,218 (81.38%)	35,892,428 (71.59%)	27,581,651 (55.01%)
VLP1	51,866,423	1,759,382 (3.39%)	40,892,763 (78.84%)	36,130,195 (69.66%)	27,677,497 (53.36%)
VLP2	50,104,549	1,496,313 (2.99%)	37,312,069 (74.47%)	32,930,422 (65.72%)	25,204,220 (50.30%)

Note: Mapped_MT: the number of reads mapped to mitochondria (the ratio in brackets is the percentage of reads mapped to mitochondria relative to clean reads). Mapped_non_MT: the number of reads mapped to regions other than mitochondria (the proportion in parentheses is the percentage of reads compared with clean reads for regions other than mitochondria). Unique_mapped: the number of reads with unique mapped positions in the reference sequence (the proportion in parentheses is the percentage of unique_mapped reads relative to clean reads). Unique_mapped_dedup: the number of reads excluding multiple Unique_mapped reads, while the mapped reads were uniquely matched to the read in the reference sequence (the proportion in parentheses is the percentage of unique_mapped_dedup reads relative to clean reads).

**Table 2:** Upregulated HOMER de novo motif analysis in the VLP group vs. the control group.

Motifs	Factors	*p*-Value	%Targets	%Background
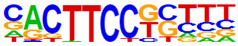	GABPA(ETS)	10^−623^	33.64%	11.44%
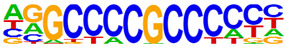	SP1	10^−581^	32.68%	11.39%
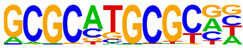	NRF1	10^−362^	14.56%	3.64%
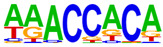	RUNX(Runt)	10^−319^	28.24%	12.53%
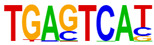	AP-1	10^−304^	17.35%	5.74%
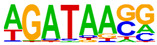	GATA3(Zf)	10^−216^	19.12%	8.22%
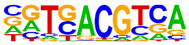	CREB1	10^−212^	21.38%	9.85%
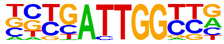	NFY(CCAAT)	10^−175^	14.46%	5.92%
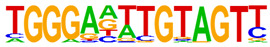	GFY	10^−159^	3.87%	0.54%
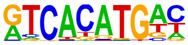	USF2	10^−91^	8.65%	3.73%
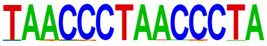	RARg(NR)	10^−83^	0.84%	0.02%
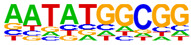	YY1(Zf)	10^−82^	4.53%	1.41%
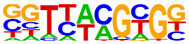	Mitf	10^−70^	16.30%	9.99%
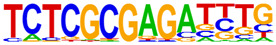	ZBTB33	10^−55^	3.40%	1.14%
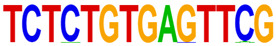	RUNX1	10^−42^	0.42%	0.01%

**Table 3:** Downregulated HOMER de novo motif analysis in the iMR-VLP group vs. the VLP group.

Motifs	Factors	*p*-Value	%Targets	%Background
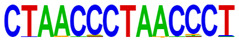	CRX	10^−84^	10.03%	0.02%
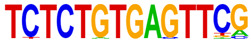	RUNX1	10^−56^	10.57%	0.16%
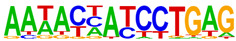	Esrra	10^−31^	12.74%	1.24%
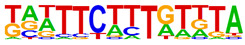	PB0166.1_Sox12_2	10^−30^	25.47%	6.47%
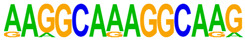	SD0002.1_at_AC_acceptor	10^−29^	11.65%	1.07%
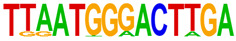	PB0135.1_Hoxa3_2	10^−27^	5.96%	0.15%
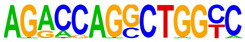	ARE(NR)	10^−25^	12.20%	1.50%
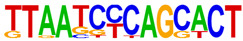	Gfi1b	10^−25^	11.65%	1.35%
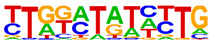	NF1:FOXA1(CTF,Forkhead)	10^−24^	15.45%	2.84%
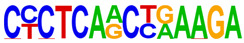	Nkx2-5(var.2)	10^−23^	3.52%	0.03%
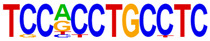	Slug(Zf)	10^−22^	12.20%	1.80%
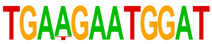	Pit1 + 1 bp(Homeobox)	10^−21^	2.98%	0.02%
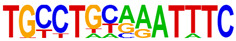	NFκB-p65-Rel(RHD)	10^−20^	4.61%	0.12%
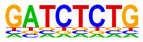	PB0126.1_Gata5_2	10^−20^	10.84%	1.57%
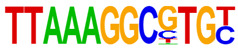	PB0180.1_Sp4_2	10^−20^	7.32%	0.60%

**Table 4:** Primers designed for genes encoding the cytokines and transcription factors.

Name	Sequence (5′-3′)	Tm (°C)	Product Size (bp)
*NF-κB*	Forward: CATCCTCGTCCGCCTATTAC	50	93
	Reverse: GACTCTCCTCCCTTTCCTTGT		
*MAPK*	Forward: AACTATTTGCTTTCTCTCCC	50	133
	Reverse: TGTTCAACTTCAATCCTCTT		
*IFN-γ*	Forward: AGACTCTCATTGCGGGGTTGTATC	54	160
	Reverse: ACAGTGTAGACATCTCCTCCCATCAG		
*TNF-α*	Forward: AAAGGGAGAGTGGTCAGGTTGC	51	95
	Reverse: CTCAGGGAAGAATCTGGAAAGGT		
*IL-9*	Forward: CAATGCCACACAGAAATCAA	55	99
	Reverse: CAGGCAGGAAAAGGACGCTT		
*IL-10*	Forward: CGCCCTATTTAGAAAGAAGCCCA	52	140
	Reverse: AAAGGAAGAACCCCTCCCATCAT		
*GADPH*	Forward: CCTTCCGTGTTCCTAC	45	152
	Reverse: GACAACCTGGTCCTCA		

## Data Availability

All the sequencing data are available in the NCBI Database and the accession number for the SRA data reported in this paper is PRJNA970980.
